# Supplementary material for: The interactome of intact mitochondria by cross-linking mass spectrometry provides evidence for coexisting respiratory supercomplexes
Source: Mol Cell Proteomics. 2017 Dec 8;17(2):216–32. doi: 10.1074/mcp.RA117.000470 (PMC5795388; doi:10.1074/mcp.RA117.000470)
Supplement: Supplemental Data [file supp_RA117.000470_133922_0_supp_23978_fzffwf.zip › spectra_annotation/mito_spectra_annotation/114-1-4-1-11-1.pdf]

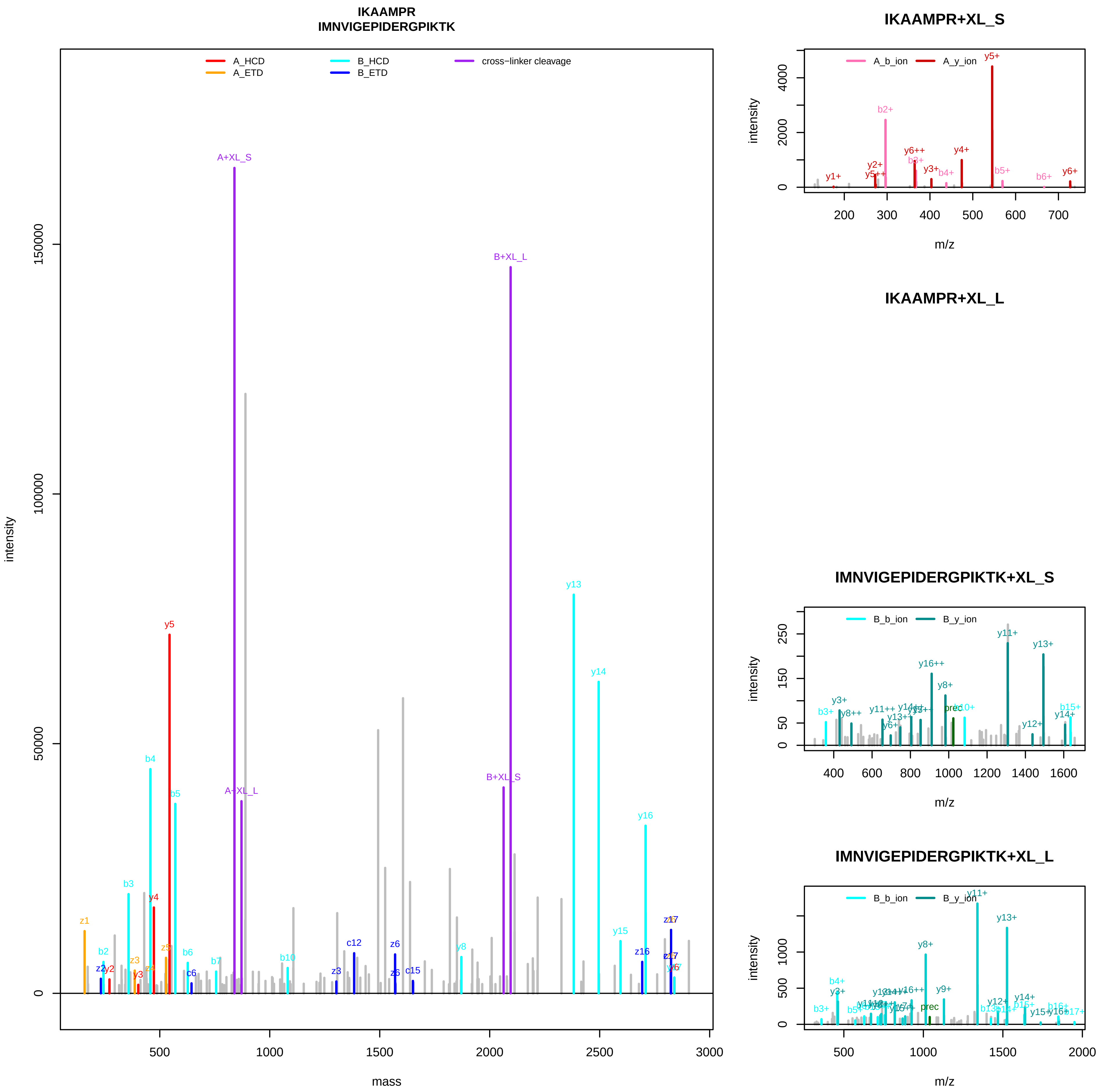

IKAAMPR
IMNVIGEPIDERGPIKTK
A_HCD
A_ETD
B_HCD
B_ETD
cross−linker cleavage
A+XL_S
B+XL_L
A+XL_L
B+XL_S
y13
y14
y16
y15
y5
y4
b4
b5
b3
b2
z1
z2
y2
z3
y3
z5
b6
c6
b7
b10
z3
c12
z6
c15
y8
z16
z17
intensity
mass
0
50000
100000
150000
500
1000
1500
2000
2500
3000
IKAAMPR+XL_S
A_b_ion
A_y_ion
y5+
b2+
y6++
y2+
y5++
y3+
y4+
b4+
b5+
b6+
y6+
y1+
intensity
m/z
0
2000
4000
200
300
400
500
600
700
IKAAMPR+XL_L
IMNVIGEPIDERGPIKTK+XL_S
B_b_ion
B_y_ion
y11+
y13+
y16++
y8+
y3+
b3+
y8++
y11++
y14++
y6+
y13++
prec
b10+
y12+
y14+
b15+
intensity
m/z
0
50
150
250
400
600
800
1000
1200
1400
1600
IMNVIGEPIDERGPIKTK+XL_L
B_b_ion
B_y_ion
y11+
y13+
y8+
b4+
y3+
b3+
b5+
y16++
y9+
prec
y12+
y14+
b15+
b16+
y15+
y16+
b17+
intensity
m/z
0
500
1000
500
1000
1500
2000
